# Supplementary material for: Causal Relationship between Aging and Anorexia Nervosa: A White-Matter-Microstructure-Mediated Mendelian Randomization Analysis
Source: Biomedicines. 2024 Aug 16;12(8):1874. doi: 10.3390/biomedicines12081874 (PMC11351342; doi:10.3390/biomedicines12081874)
Supplement: Supplementary file 1 [file biomedicines-12-01874-s001.zip › Supplementary Figure/Figure S8/Leaveoneout plot of MR analysis for TL to ubm-b-1602.pdf]

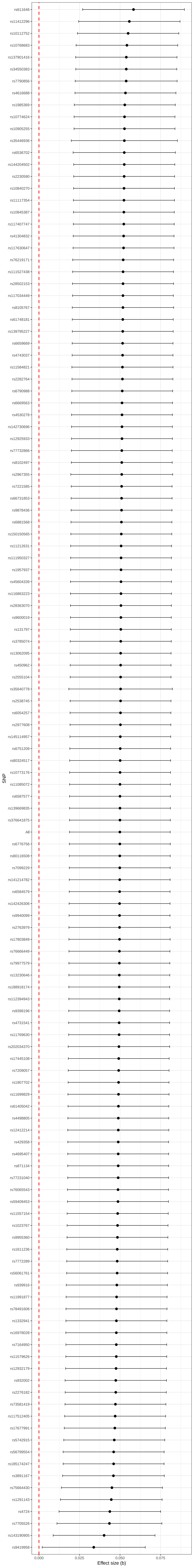
SNP
rs611646
rs11412296
rs10112752
rs10768683
rs137901416
rs34550383
rs7790856
rs4616688
rs1985369
rs10774624
rs10905255
rs35446936
rs6536702
rs144204502
rs2230590
rs10840270
rs11117354
rs10845387
rs117407747
rs41304832
rs117630647
rs76219171
rs111527438
rs28502153
rs117034449
rs8105767
rs61748181
rs139795227
rs6659669
rs4743037
rs11584821
rs2282764
rs6790988
rs6669563
rs4530278
rs142730696
rs12925933
rs77732866
rs8102497
rs2967355
rs7221585
rs66731853
rs9878436
rs6881568
rs150150565
rs11212631
rs111950327
rs1957937
rs45604339
rs116863223
rs28363070
rs9600019
rs131797
rs3785074
rs13062095
rs450962
rs2555104
rs35640778
rs2538745
rs6054257
rs2977608
rs145114957
rs6751209
rs80324517
rs10773176
rs11085072
rs6587577
rs139669835
rs376641875
A8
rs6776756
rs80116508
rs7099229
rs141214782
rs6584579
rs142426306
rs9940099
rs2763979
rs17803849
rs76666449
rs79977579
rs12230646
rs188918174
rs112394943
rs9398196
rs4731541
rs11769630
rs202034370
rs17445108
rs7209057
rs1907702
rs11699829
rs61405042
rs4498805
rs12412214
rs429358
rs4695407
rs871134
rs77231040
rs76065543
rs59409453
rs11557154
rs1023767
rs9955360
rs1611236
rs7772289
rs56061761
rs939916
rs11991877
rs78491606
rs1332941
rs16978028
rs7164950
rs11579626
rs12932179
rs932002
rs2276182
rs73581419
rs117512405
rs17677991
rs5742915
rs56799554
rs185174247
rs3891167
rs75664430
rs1291143
rs4724
rs7705526
rs143190905
rs9419958
0.000
0.025
0.050
0.075
Effect size (b)
